# Correction of Huntington’s Disease Phenotype by Genistein-Induced Autophagy in the Cellular Model

**DOI:** 10.1007/s12017-018-8482-1

**Published:** 2018-02-12

**Authors:** Karolina Pierzynowska, Lidia Gaffke, Aleksandra Hać, Jagoda Mantej, Natalia Niedziałek, Joanna Brokowska, Grzegorz Węgrzyn

**Affiliations:** Department of Molecular Biology, University of Gdańsk, Wita Stwosza 59, 80-308 Gdańsk, Poland

**Keywords:** Huntington’s disease, Genistein, Autophagy, Protein aggregates

## Abstract

Huntington’s disease (HD) is a monogenic disorder, caused by mutations in the *HTT* gene which result in expansion of CAG triplets. The product of the mutated gene is misfolded huntingtin protein that forms aggregates leading to impairment of neuronal function, neurodegeneration, motor abnormalities and cognitive deficits. No effective cure is currently available for HD. Here we studied effects of genistein (trihydroxyisoflavone) on a HD cellular model consisting of HEK-293 cells transfected with a plasmid bearing mutated *HTT* gene. Both level of mutated huntingtin and number of aggregates were significantly decreased in genistein-treated HD cell model. This led to increased viability of the cells. Autophagy was up-regulated while inhibition of lysosomal functions by chloroquine impaired the genistein-mediated degradation of the mutated huntingtin aggregates. Hence, we conclude that through stimulating autophagy, genistein removes the major pathogenic factor of HD. Prolonged induction of autophagy was suspected previously to be risky for patients due to putative adverse effects; however, genistein has been demonstrated recently to be safe and suitable for long-term therapies even at doses as high as 150 mg/kg/day. Therefore, results presented in this report provide a basis for the use of genistein in further studies on development of the potential treatment of HD.

## Introduction

Neurodegenerative diseases will pose an increasing burden on society. Most of them are incurable, and despite extensive work and many efforts, no effective treatment can be proposed to a vast majority of patients (Pritchard et al. 2013). Among these diseases, some are caused by accumulation of protein aggregates and resultant loss of neuronal functions. Huntington’s disease (HD) is an example of such disorders, which can also be considered as a model of them due to its well-defined genetic cause (it is a monogenic disease) and pathomechanism at the cellular level (Morreale 2015).

HD is a monogenic disease inherited in an autosomal dominant manner. The genetic defect consists of expansions of CAG repeats in exon 1 of the *HTT* gene, coding for the huntingtin protein. This results in appearance of long series of glutamine residues in the huntingtin protein, known as the polyQ tract. American College of Medical Genetics and the American Society of Human Genetics recommend following classification of HD, based on the number of CAG repeats: under 27 repeats, unequivocally normal; 27–35 repeats, normal but may expand in future generations; 36–39 repeats, abnormal but associated with reduced penetrance; 40 or more repeats, abnormal with full penetrance. Mutant huntingtin protein tends to form aggregates that are hardly degradable and became a distinctive feature of Huntington’s disease. Its main clinical symptoms can include movement, cognitive and psychiatric disturbances. Unfortunately, the disease is fatal and most of patients die within 15–20 years from diagnosis (Morreale 2015). Currently, there is no effective treatment of Huntington’s disease, and its management focuses only on relieving the symptoms (Shannon and Fraint 2015). There were various approaches to inhibit expression of the mutated *HTT* allele, inactivation of the mutated huntingtin, and improvement of cell viability (Shannon and Fraint 2015). Nevertheless, decreasing the level of mutated huntingtin is considered as one of the most promising possibilities to find an efficient therapy for HD (Aronin and DiFiglia 2014). Stimulation of protein degradation appears an obvious option for such an approach.

In human cells, there are two potential degradation pathways of proteins, including huntingtin. One of them consists of the proteasomes, where ubiquitin-labeled proteins are directed to the degradation system (so-called ubiquitin–proteasome system or UPS). The second pathway is based on the autophagy process, in which lysosomal hydrolases degrade damaged organelles and macromolecules sequestered by autophagosomes. It was demonstrated that in HD cells, there are inappropriate interactions between ubiquitin and the proteasome complex which results in impairment of UPS (Jana et al. 2001; Bennett et al. 2007; Finkbeiner and Mitra 2008). Contaminations of proteasomal fragments were found in huntingtin aggregates, suggesting that the latter structures might affect UPS (Gil and Rego 2008). Therefore, putative stimulation of these systems appears unlikely as a method for treatment of HD. Instead, it is postulated that UPS may be involved in degradation of normal huntingtin while the mutant form is directed to the lysosomal pathway (Ravikumar et al. 2002, 2004; Bhutani et al. 2007).

There are many known chemicals acting as autophagy stimulators. However, none of them have been successfully used as a therapeutic molecule since they are either toxic or unable to cross the blood–brain-barrier (Gros and Muller 2014; Yang et al. 2013). On the other hand, recent studies performed in our laboratory indicated that genistein (trihydroxyisoflavone or 5, 7-dihydroxy-3- (4-hydroxyphenyl)-4*H*-1-benzopyran-4-one), a natural isoflavone, stimulates expression of TFEB, a transcription factor which is a master stimulator of lysosomal biogenesis (Moskot et al. 2014). Moreover, genistein-mediated negative regulation of mTOR caused enhanced dephosphorylation and subsequent translocation of TFEB into cell nuclei. Thus, significant increase in lysosomal content and activity was observed in genistein-treated cells (Moskot et al. 2015a). Since genistein can cross the blood–brain-barrier (Tsai 2005), and it was demonstrated to be safe when used for a long time (over 1 year) at concentration as high as 150 mg/kg/day during a clinical trial (Kim et al. 2013), we aimed to test effects of this isoflavone on mutated huntingtin in HEK-293 cells expressing the *HTT* gene exon 1 with 74 CAG repeats which is a commonly used cellular model of HD.

## Materials and Methods

### Reagents

Genistein (99% purity; #446-72-0), purchased from Pharmaceutical Research Institute in Warsaw (Poland), was diluted in DMSO at stock concentrations of 30, 60, and 100 mM and stored at − 20 °C. Plasmid pEGFP (encoding EGFP) was from Addgene (#6077-1), as were plasmids pEGFP-Q74 (encoding EGFP protein fused to a fragment of huntingtin corresponding to the exon 1 with 74 CAG repeats; c.54GCA[74]; Addgene; #40262) and pEGFP-Q23 (encoding EGFP protein fused to a fragment of huntingtin corresponding to the exon 1 with 23 CAG repeats; c.54GCA[23]; Addgene; #40263) (these plasmids were gifts from Dr. David Rubinsztein to Addgene). Antibodies against GFP (used at dilution 1:4000) were from Santa Cruz Biotechnology (#sc-9996), and those against LC3 (used at dilution 1:2000) from MBL International (#PM036). The anti-mouse secondary antibodies (used at dilution 1:4000), as well as anti-β-actin antibodies (used at dilution 1:25,000) conjugated with HRP (#A3854), were from Sigma-Aldrich. Lysotracker Red was purchased from Life Technologies (#L-7528). Muse^®^ Annexin V and Dead Cell Assay Kit were purchased from Merck (#MCH100105).

### Cell Lines and Cell Cultures

Immortalized human embryonic kidney (HEK-293) cells were obtained from ATCC (#CRL-1537). They were maintained in the DMEM medium containing 10% fetal bovine serum and the penicillin–streptomycin mixture. Cells were cultured at 37 °C in a humidified atmosphere with 5% CO_2_.

### Transient Transfection with Plasmid DNA

HEK-293 cells were transfected with vectors encoding EGFP, EGFP-Q23 or EGFP-Q74 using TurboFect, according to the manufacturer’s instructions. Following 6-h treatment, the medium was exchanged with a new one, and cells were treated with DMSO (control cells) or 30, 60 or 100 µM genistein for 48 h at 37 °C. In samples with lysosome inhibition, chloroquine was added to final concentration of 10 μM for 1 h before the genistein/DMSO treatment. The average efficiency of transfection, calculated for all plasmids (as it was very similar in all cases), was 51 ± 6%.

### Fluorescence Microscopy

2.5 × 10^5^ HEK-293 cells were passaged on coverslips in each well of 6-well plate, and allowed to attach overnight. Cells were then transfected and treated with DMSO (control cells) or 30, 60 or 100 µM genistein for 48 h at 37 °C. In samples with lysosome inhibition, chloroquine was added to final concentration of 10 μM for 1 h before the genistein/DMSO treatment, and Lysotracker Red was added to final concentration of 100 nM for 1 h before sample withdrawal. The cells were fixed with 2% paraformaldehyde in phosphate buffered saline (PBS), rinsed with PBS, and incubated with DAPI fluorescence dye. Coverslips were adhered to glass slides with a mounting medium, and at the next day, they were observed under the Nikon Eclipse E800 microscope. Number and size of aggregates, as well as number of lysosomes, were assessed manually (numbers were determined by counting aggregates/lysosomes in at least 100 transfected cells, and sizes of aggregates were measured under the microscope with the software provided by the microscope manufacturer); each assessment was performed by at least two independent assessors (KP, JM and/or NN). For observation of lysosomes in living cells, the procedure was analogous, but the cells were not fixed with 2% paraformaldehyde and staining with DAPI was omitted; instead, after treatment with Lysotracker Red, the cells were washed 3 times with PBS, a drop of cell suspension was put between two coverslips, and the cells were observed and photographed under the Nikon Eclipse E800 microscope with and without yellow filter.

### Immunoblotting

7.5 × 10^5^ HEK-293 cells were passaged on plates (6 cm in diameter), and allowed to attach overnight. Cells were then transfected, and treated with DMSO (control cells) or 30, 60 or 100 µM genistein for 48 h at 37 °C. In samples with lysosome inhibition, chloroquine was added to final concentration of 10 μM for 1 h before the genistein/DMSO treatment. Next, cells were lysed with a solution containing 1% Triton X-100, 0.5 mM EDTA, 150 mM NaCl, 50 mM Tris, pH 7.5, and a mixture of protease and phosphatase inhibitors (Roche Applied Science, #05892791001 and #11873580001). The lysates were cleared by centrifugation; proteins were separated by SDS-PAGE and transferred to a PVDF membrane overnight. The membrane was blocked with 5% non-fat dry milk in PBST buffer and then incubated with primary antibodies overnight at 4 °C. At the next day, the membrane was incubated with secondary antibodies at room temperature for 1 h, treated with a solution of substrates for detecting of HRP, and exposed to the X-ray film. The intensities of bands were analyzed with the QuantityOne software. Since aggregates of huntingtin do not enter the separating gel but they remain on top of the lane in the stacking gel, they were detected after transferring the material from the latter gel onto the PVDF membrane. Soluble huntingtin was analyzed from the separating gel. To quantify the amounts of both forms of huntingtin, β-actin was used as an internal control, and the results were normalized relative to the β-actin level.

### MTT Cell Viability Assay

4 × 10^3^ HEK-293 cells were passaged in each well of 96-well plate, and allowed to attach overnight. Cells were then transfected, and treated with DMSO (control cells) or 30, 60 or 100 µM genistein at 37 °C. After 48 h incubation, 25 µl of MTT solution (4 mg/ml) was added to each well. Following 3-h incubation at 37 °C, formazan crystals, formed in living cells, were dissolved in 100 µl of DMSO. Absorbance was measured at 570 nm and 620 nm (reference wavelength) in a Victor^3^ microplate reader.

### Annexin V and Dead Cell Assay

1.5 × 10^5^ HEK-293 cells were passaged in each well of 12-well plate, and allowed to attach overnight. Cells were then transfected, and treated with DMSO (control cells) or 30, 60 or 100 µM genistein at 37 °C. After 48-h incubation, the cells were trypsinized, transferred to a tube, centrifuged and suspended in a new medium. Then, the number of live, apoptotic and dead cells was measured using the Muse^®^ Annexin V and Dead Cell Assay Kit (Merck).

### Approval and Accordance

All experimental protocols were approved by the Head of Department of Molecular Biology and Dean of Faculty of Biology, according to the procedures described in guidelines and regulations of the Vice-rector for Research of University of Gdańsk.

## Results

HEK-293 cell line transfected with plasmids expressing either no *HTT* gene (pEGFP), wild-type exon 1 of the *HTT* gene (pEGFP-Q23; with c.54GCA[23]) or mutant exon 1 of the *HTT* gene (pEGFP-Q74; with c.54GCA[74]) was employed in this study. We have chosen this model as the aim of this work was to assess effects of genistein on mutated huntingtin that accumulates in cells. The plasmid expressing the c.54GCA[74] variant of *HTT* ensures high level production of huntingtin with a high number of Q repeats. This allows unambiguous determination of the metabolism of the mutated protein and accurate assessment of effects of tested compounds.

Employing the HEK-293 cell line transfected with either vector or wild-type or the mutated *HTT* gene fragment, we have found that following relatively short (48 h) treatment with genistein, the level of both aggregates and the soluble form of the mutated gene product were significantly decreased relative to untreated cells (DMSO, used as a control), and the effects were within the dose–response correlation (Fig. 1). No significant differences in the levels of wild-type huntingtin (only soluble form, as no aggregates could be detected) were observed at all tested genistein concentrations. Furthermore, microscopic analyses indicated that number and size of mutated huntingtin aggregates decreased considerably under these conditions (Fig. 2). In control experiments, uniform distribution of wild-type huntingtin in cells was observed (Fig. 2). These results indicate that genistein treatment results in a significant reduction in mutated huntingtin in the HD cell model, while having no considerable effects on the level of the wild-type protein.

**Fig. 1:**
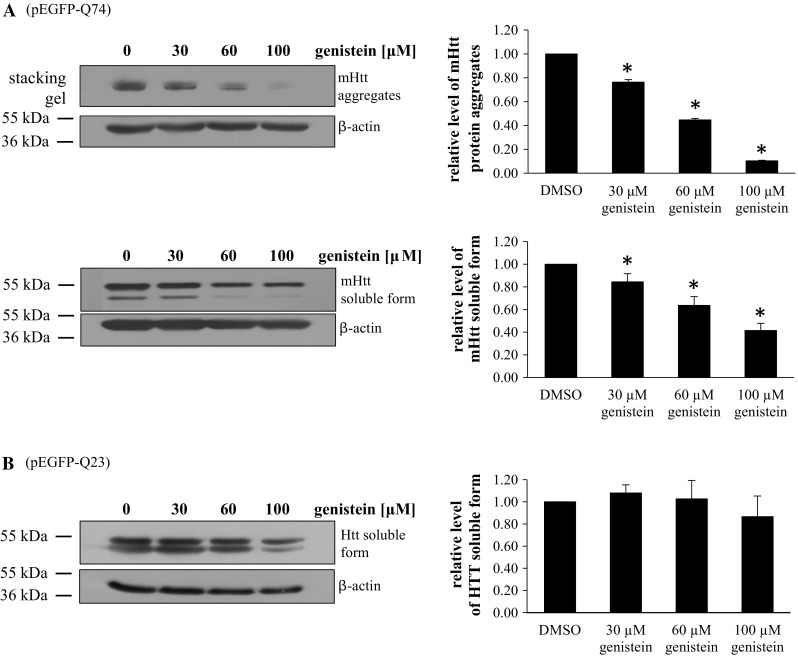
Reduction in the level of mutated huntingtin (mHTT) by genistein in the HD cellular model. HEK-293 cells were transfected with plasmids encoding EGFP-Q74 (EGFP protein fused to a fragment of huntingtin corresponding to the exon 1 with 74 CAG repeats; panel **a**) or EGFP-Q23 (EGFP protein fused to a fragment of huntingtin corresponding to the exon 1 with 23 CAG repeats; panel **b**), and treated with indicated concentrations of genistein for 48 h. Levels of mHTT (aggregates and soluble forms, panel **a**) or HTT (soluble form only, as no aggregates could be detected, panel **b**) were assessed by Western blotting with anti-GFP antibodies and densitometry (in the soluble form, the lower band is a GFP partial degradation product which was not considered in quantitative analysis), with normalization against β-actin. Quantification of results exemplified by representative blots is shown on histograms near corresponding pictures. The results are mean values from 3 independent experiments with error bars representing SD. Statistical significance (*p* < 0.05 in the *t*-Student test) of differences between results obtained in tested samples and controls is indicated by asterisk

**Fig. 2:**
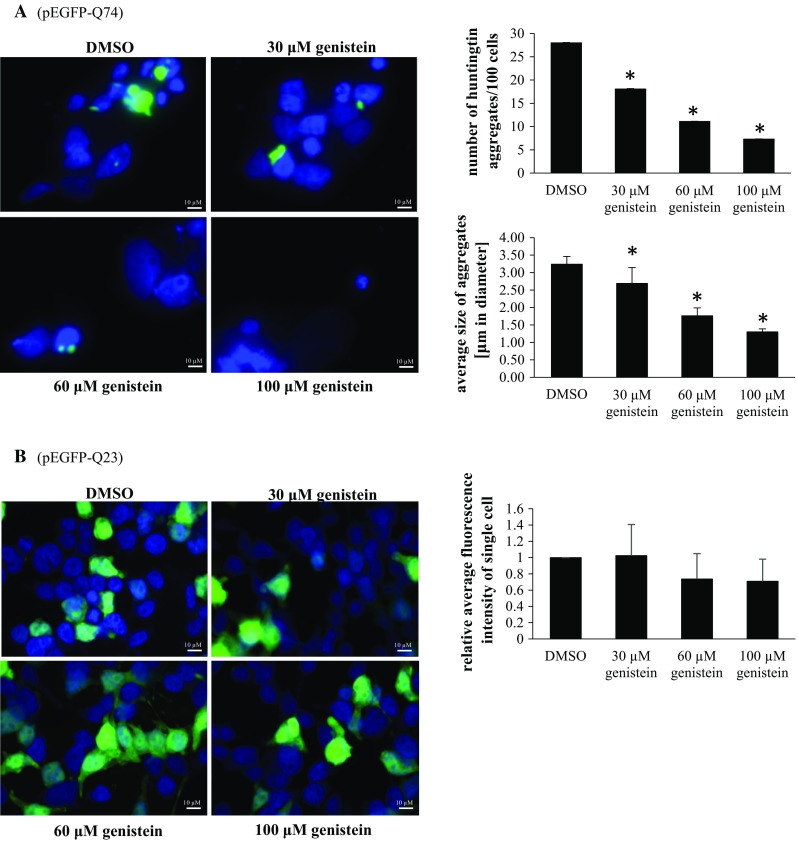
Reduction in the number and size of aggregates of mutated huntingtin (mHTT) by genistein in the HD cellular model. HEK-293 cells were transfected with plasmids encoding either EGFP-Q74 (EGFP protein fused to a fragment of huntingtin corresponding to the exon 1 with 74 CAG repeats; panel **a**) or EGFP-Q23 (EGFP protein fused to a fragment of huntingtin corresponding to the exon 1 with 23 CAG repeats; panel **b**), and treated with indicated concentrations of genistein for 48 h. Number and size of aggregates (visible as sharp fluorescent foci in panel **a**, in contrast to fuzzy fluorescent areas, dispersed throughout the whole cell and corresponding to non-aggregated, soluble proteins, visible in panel **b**) were determined by fluorescent microscopy and densitometry. Quantification of results exemplified by representative microphotographs is shown on histograms near corresponding pictures. The results are mean values from 3 independent experiments with error bars representing SD. Statistical significance (*p* < 0.05 in the *t*-Student test) of differences between results obtained in tested samples and controls is indicated by asterisk

To test global effects of genistein on HD cells’ physiology, we assessed cell viability by using the MTT assay. Metabolic activity (being an indicator of cell viability in this assay) of HD cells was significantly decreased relative to control cells transfected with the plasmid expressing wild-type *HTT* allele (Fig. 3a). However, treatment of HD cells with genistein restored their viability (Fig. 3a). This also demonstrated that genistein is not toxic for cells, even at relatively high concentrations. In control experiments, we aimed to demonstrate deleterious effects of a known cytotoxic compound, and H_2_O_2_ was highly toxic to the cells, as expected. Nevertheless, it was plausible that lower number of HD cells (Fig. 3a) resulted from slower proliferation of cells rather than from lower viability. Therefore, we have performed the Annexin V assay. No increase in a fraction of apoptotic cells treated with genistein was observed in cells expressing wild-type *HTT* allele (Fig. 3b), indicating again a lack of cytotoxicity of this compound. In the HD model, the fraction of apoptotic cells was significantly higher, over 30%, but decreased considerably after treatment with genistein (Fig. 3b). These results indicate that genistein is not cytotoxic, but has a positive effect not only on the reduction in number and size of aggregates and the level of mutated huntingtin, but also on the whole cell physiology.

**Fig. 3:**
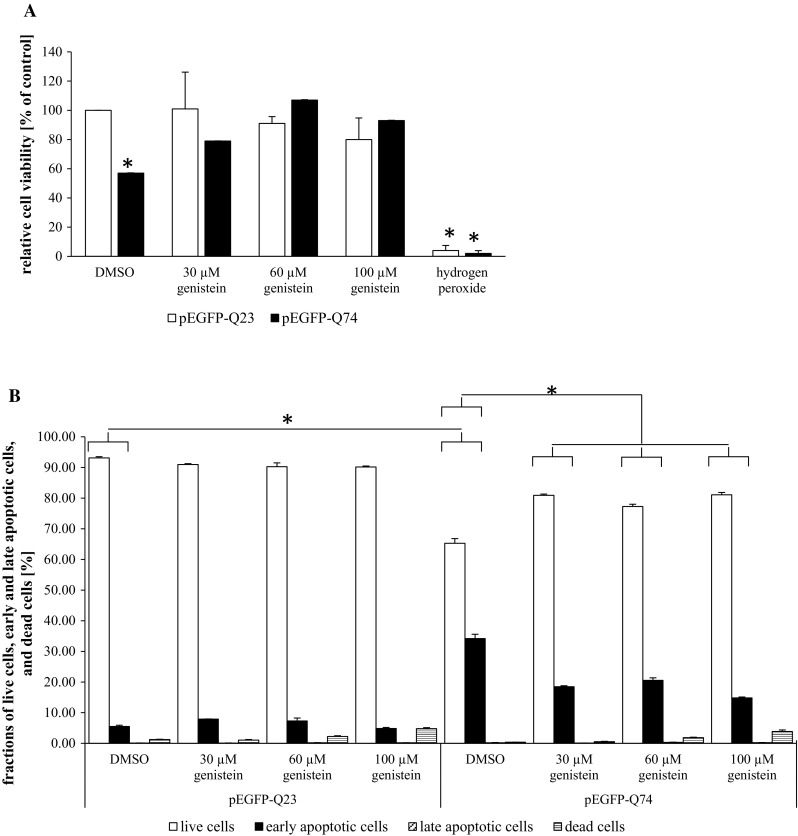
Genistein is not cytotoxic and restores viability of the HD cellular model. MTT (**a**) and Annexin V and dead cell (**b**) assays. In the MTT test (**a**), HEK-293 cells were transfected with plasmids encoding either EGFP-Q23 (pEGFP-Q23; open columns) or EGFP-Q74 (pEGFP-Q74, closed columns), and treated with indicated concentrations of genistein for 48 h. In control experiments, the cells were treated with 2.5 μM hydrogen peroxide. Viability of cells was assessed by the MTT test. The results are mean values from 3 independent experiments with error bars representing SD. Statistical significance (*p* < 0.05 in the *t*-Student test) of differences between results obtained in tested samples and controls (cells transfected with pEGFP-Q23, without genistein) is indicated by asterisk. Experiments shown in panel **b** were performed analogously, but Annexin V and dead cell assay was performed to measure fractions of live, early apoptotic, late apoptotic, and dead cells. Statistical significance (*p* < 0.05 in the *t*-Student test) of differences between results obtained in indicated samples is shown by asterisk

To learn whether genistein can induce lysosomal biogenesis and the autophagy process in HD, we monitored the number and size of lysosomes, and the level of the LC3-II protein (the marker of autophagy) in genistein-treated HD and control cells. Addition of this isoflavone to cell cultures caused a significant increase in the amount of LC3-II irrespective of the *HTT* allele; however, the induction of autophagy was more pronounced in cells expressing the mutated *HTT* gene (Fig. 4). Stimulation of lysosomal biogenesis was also evident under these conditions in cells expressing c.54GCA[74] or c.54GCA[23] from plasmid pEGFP-Q74 or pEGFP-Q23, respectively (Fig. 5).

**Fig. 4:**
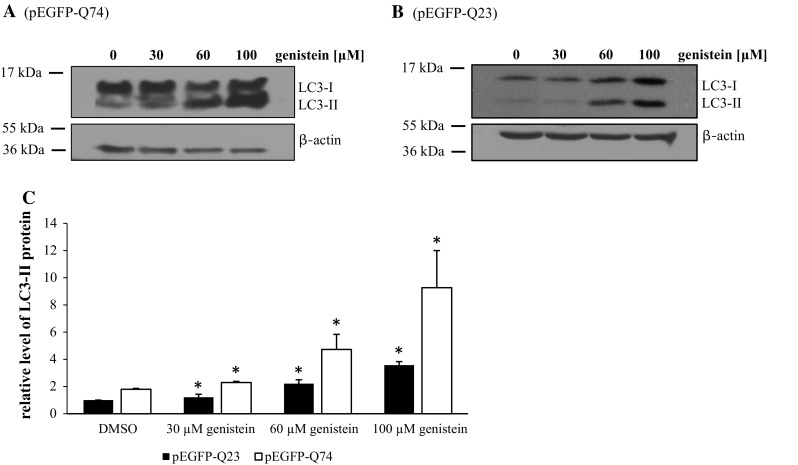
Genistein induces the autophagy process in the HD cellular model. HEK-293 cells were transfected with plasmids encoding EGFP-Q74 (panel **a**) or EGFP-Q23 (panel **b**) and treated with indicated concentrations of genistein for 48 h. Levels of the LC3 protein, including the LC3-II form, being the autophagy marker, were assessed by Western blotting and densitometry, with normalization against β-actin. Quantification of results exemplified by representative blots is shown on panel **c** (closed columns for experiments with pEGFP-Q23, and open columns for experiments with pEGFP-Q74). The results are mean values from 3 independent experiments with error bars representing SD. Statistical significance (*p* < 0.05 in the *t*-Student test) of differences between results obtained in particular experiment (samples with genistein versus controls with the same plasmid but without genistein) is indicated by asterisk. When results of experiments with pEGFP-Q23 and pEGFP-Q74 were compared, statistically significant differences (*p* < 0.05 in the *t*-Student test) were found for each pair represented by closed and open columns (control cells and cells treated with all tested concentrations of genistein)

**Fig. 5:**
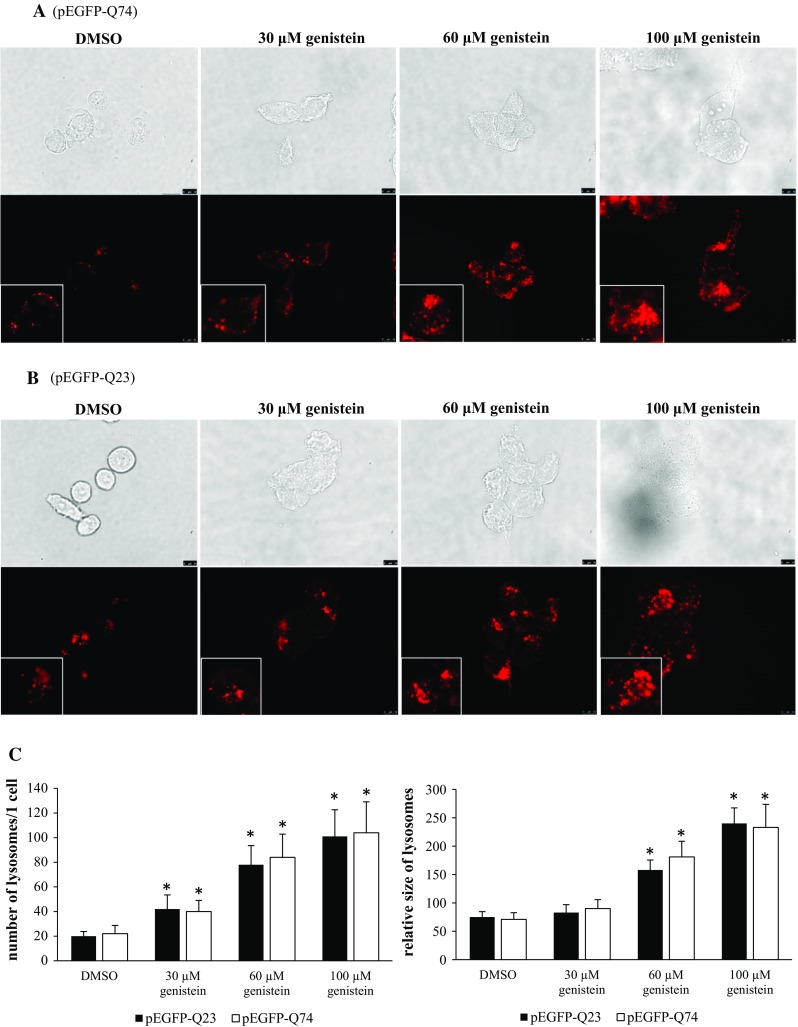
Genistein stimulates lysosomal biogenesis in the HD cellular model. HEK-293 cells were transfected with plasmids encoding either EGFP-Q74 (panel **a**) or EGFP-Q23 (panel **b**), and treated with indicated concentrations of genistein for 48 h. Number and size of lysosomes were determined by fluorescent microscopy analysis of living cells treated with Lysotracker Red, followed by densitometry. The pictures were taken without (upper photographs) and with (lower photographs) yellow filter (upper panels indicate that similar number of cells was present in every picture; in lower panels, fragments of photographs are enlarged and shown in a lower left corner of each panel to depict single lysosomes more accurately). Quantification of results exemplified by representative pictures is shown on panel **c** (closed columns for experiments with pEGFP-Q23, and open columns for experiments with pEGFP-Q74). The results are mean values from 3 independent experiments with error bars representing SD. Statistical significance (*p* < 0.05 in the one way ANOVA test) of differences between results obtained in particular experiment (samples with genistein versus controls with the same plasmid but without genistein) is indicated by asterisk. When results of experiments with pEGFP-Q23 and pEGFP-Q74 were compared, no statistically significant differences (*p* > 0.05 in the one way ANOVA test) could be find for any pair represented by closed and open columns (control cells and cells treated with all tested concentrations of genistein)

We asked whether the autophagy induction is responsible for the observed changes in the level of mutated huntingtin and number of aggregates. Thus, lysosomal functions were partially blocked by the use of low concentration (10 μM) of chloroquine (higher concentrations were highly deleterious to cells, making the results hardly interpretable), and the effects of genistein were assessed. Inhibition of lysosomes (confirmed by an increase in the level of LC3-II (Fig. 6) which could not be degraded due to chloroquine-mediated increase in intralysosomal pH and resultant impairment of lysosomal enzymes’ activities (see Tasdemir et al. 2008, Mizushima et al. 2010)) resulted in more efficient accumulation of mutated huntingtin and less pronounced effects of the isoflavone on this protein (Figs. 6, 7). Chloroquine caused a strong impairment of genistein-mediated stimulation of mHTT degradation at 30 μM concentration of this isoflavone, while it was significantly less pronounced at higher genistein concentrations (Fig. 6). Therefore, we conclude that genistein treatment causes the correction of the HD phenotype in the cell model, predominantly due to induction of the autophagy process, while at higher concentrations of this isoflavone (60 and 100 μM) other, as yet unidentified, process(es) leading to degradation of mutant huntingting might also be activated. Intriguingly, alleviation of genistein-mediated disappearance of mutated huntingtin by chloroquine was more pronounced in experiments with observation of the aggregates under microscope (Fig. 7) than in determination of soluble protein levels by Western blotting (Fig. 6). This suggests that autophagy may be predominant in destruction of the aggregates, while other processes might be additionally involved in degradation of the soluble form, particularly at higher concentrations of genistein.

**Fig. 6:**
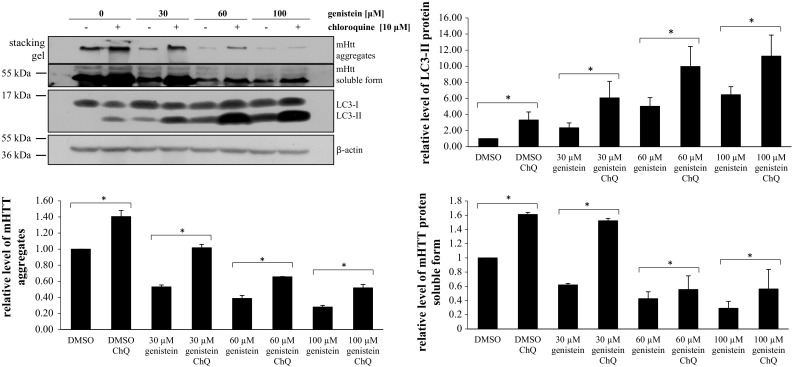
Inhibition of lysosomal functions impairs effects of genistein on levels of mutated huntingtin (mHTT). HEK-293 cells were transfected with plasmid encoding EGFP-Q74 (EGFP protein fused to a fragment of huntingtin corresponding to the exon 1 with 74 CAG repeats) and treated with indicated concentrations of genistein for 48 h. When indicated, chloroquine was added to final concentration of 10 μM, 60 min before addition of genistein. Levels of mHTT aggregates and soluble form, and those of the LC3-II protein, were assessed by Western blotting (with anti-GFP and anti-LC3 antibodies, respectively) and densitometry, with normalization against β-actin. Quantification of results exemplified by representative blots is shown in histograms below corresponding pictures (note that due to large differences in intensities between particular bands, different exposure times had to be used to perform densitometry properly). The results are mean values from 5 independent experiments with error bars representing SD. Statistical significance (*p* < 0.05 in the *t*-Student test) of difference between results of marked experiments is indicated by asterisk

**Fig. 7:**
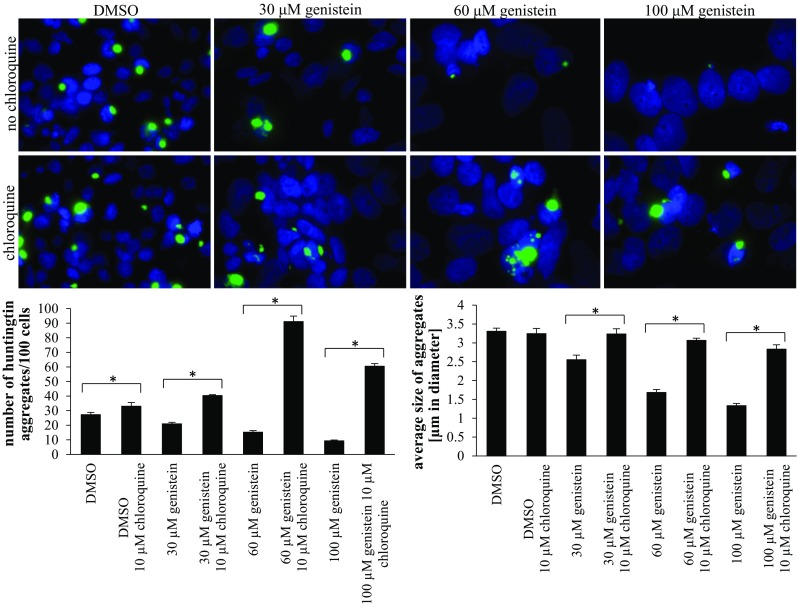
Inhibition of lysosomal functions impairs effects of genistein on number and size of mutated huntingtin (mHTT) aggregates. HEK-293 cells were transfected with plasmids encoding EGFP-Q74 (EGFP protein fused to a fragment of huntingtin corresponding to the exon 1 with 74 CAG repeats) and treated with indicated concentrations of genistein for 48 h. When indicated, chloroquine was added to final concentration of 10 μM, 60 min before addition of genistein. Number and size of aggregates were determined by fluorescent microscopy and densitometry. Quantification of results exemplified by representative pictures is shown in histograms below corresponding pictures. The results are mean values from 3 independent experiments with error bars representing SD. Statistical significance (*p* < 0.05 in the *t*-Student test) of difference between results of marked experiments is indicated by asterisk

## Discussion

The results presented in this report indicate that genistein corrects the HD phenotype in cellular model of this disease (Figs. 1, 2, 3), predominantly by activating the autophagy process (Figs. 4, 5, 6, 7). Autophagy appears to be the major, if not the only, process responsible for mutant huntingtin degradation in genistein-treated HD cells at relatively low (30 μM) concentration of this isoflavone (Fig. 6). At higher genistein concentrations (60 and 100 μM), degradation of mutant huntingtin still occurred, though less efficiently, when autophagy was blocked by chloroquine (Fig. 6). On the other hand, inhibition of destruction of huntingtin aggregates by chloroquine was still very efficient even at high genistein levels (Fig. 7). This may suggest that high genistein concentrations might cause activation of other, as yet unidentified, process(es) leading to degradation of soluble mutant huntingtin. However, it is worth noting that it is unlikely to achieve 60 or 100 μM concentrations of genistein in vivo (in animal or human bodies); thus, results obtained with 30 μM genistein appear more relevant in the light of a possible use of this isoflavone as a therapeutic.

Although autophagy can be induced also by other compounds, apart from genistein, a potential drug for treatment of a neurodegenerative genetic disease must cross the blood–brain-barrier, be non-toxic, and be suitable for a long-term administration. Such features (required to be present all together) are not met by vast majority of these substances (Yang et al. 2013). Particularly, long-term induction of autophagy was suggested as a potentially risky therapeutic option due to a probability of destruction of various cell compounds (Yang et al. 2013). Thus, any putative autophagy-stimulating drug should act gently, rather than strongly, to avoid severe adverse effects, which makes the list of such compounds very short if existing at all. On the other hand, genistein has been proposed previously as a potential drug for treatment of some genetic diseases, including cystic fibrosis and mucopolysaccharidoses, though different properties of this isoflavone (correction of the mutated CFTR protein and impairment of glycosaminoglycan synthesis, respectively) were considered (Węgrzyn et al. 2010). Quite surprisingly, another property of genistein, stimulation of lysosomal biogenesis, appeared to be potentially beneficial in treatment of mucopolysaccharidoses, a group of lysosomal storage diseases (Piotrowska et al. 2006; Jakóbkiewicz-Banecka et al. 2009). In fact, genistein was perhaps the first chemical which caused a complete correction of behavior in studies on an animal model of a neurodegenerative metabolic disease (Malinowska et al. 2010). This isoflavone is able to cross the blood–brain-barrier (Tsai 2005), and it was found to be non-toxic to animals even when used at as high concentration as 160 mg/kg/day (Malinowska et al. 2009). Subsequent clinical studies, performed with children, demonstrated its safety in the long-term (over 1 year) use at the high (150 mg/kg/day) dose (Kim et al. 2013). Currently, a phase III, double-blinded, randomized, placebo-controlled clinical trial of high dose oral genistein aglycone in patients with Sanfilippo syndrome (mucopolysaccharidosis type III) is ongoing (EudraCT number: 2013-001479-18). Thus, we suggest that genistein may be a unique candidate for an effective and safe autophagy-inducing drug.

Demonstration that genistein-mediated stimulation of autophagy causes the disappearance of mutated huntingtin aggregates in HD cells (Figs. 1, 6) may open a new possibility of treatment of this disease. Although our results do not exclude a possibility that other pathway(s) is/are stimulated by this isoflavone, particularly at high concentrations, it appears that autophagy is an effective process responsible for reduction in the protein aggregates in HD cells. In fact, autophagy highly predominates in mutant huntingtin degradation at concentration of genistein (30 μM) which is relevant in the light of its potential use as a therapeutic (Fig. 6). Because aggregates of mutant huntingtin are pathogenic factors, and since genistein fulfills all the requirements for a potential drug to treat patients with metabolic neurodegenerative diseases for a long period, it is reasonable to propose further studies on the use of this isoflavone as an anti-HD drug. Although transcripts containing translated CAG repeats play an auxiliary role in pathogenesis of HD (Galka-Marciniak et al. 2012), mutated huntingtin aggregates are the major cause of neurodegeneration (Zhao et al. 2016). Furthermore, it is worth to remind that protein aggregates are also major factors causing other neurodegenerative disorders, including Alzheimer’s disease and Parkinson’s disease (Goloubinoff 2016). Therefore, it is tempting to suggest that genistein-induced autophagy might be effective in reduction of aggregates of β-amyloid, hyper-phosphorylated τ protein, synuclein and/or parkin, which accumulate in these diseases. If this hypothesis is true, development of effective treatments for a large group of severe, neurodegenerative disorders might be possible. No cure is currently available for vast majority of such diseases, and only alleviation of symptoms and palliative care can be offered to patients.

We are aware that the model HD cells used in this work, HEK-293 cells transfected with a plasmid expressing the 1st exon of the *HTT* gene with 74 CAG repeats, do not represent neurons which are primarily affected in this disease in humans. Nevertheless, this cell line provides a useful model for investigation of various cellular mechanisms of processes that are characteristic for many cell types, including neurons. In fact, the HEK-293 cell line was successfully used in studies on different neurological diseases (Haas et al. 2014; Lecca et al. 2015; Petrosino et al. 2015), including HD (Pryor et al. 2014; Ratovitski et al. 2015; Bañez-Coronel et al. 2015; Sameni et al. 2016). Such studies can provide molecular bases for further investigations on development of specific treatment procedures and specific drugs.

Although genistein improved significantly the HD phenotype in a cellular model of this disease, there were some side effects observed in our experiments. Namely, at higher genistein concentrations (particularly 100 μM), average size of cellular nuclei increased relative to control cells (without genistein). This was observed in experiments with both pEGFP-Q23 and pEGFP-Q74 (Fig. 2), indicating that such a phenomenon occur irrespective of the form of huntingtin. We speculate that this might arise from the recently reported effects of genistein on the cell cycle (Moskot et al. 2015b). Nevertheless, documented safety of high doses of genistein in both animal studies (Malinowska et al. 2010) and clinical trial (Kim et al. 2013) indicates that this phenomenon, observed in vitro, perhaps does not affect physiology of organisms treated with this isoflavone. In fact, a lack of cytotoxic effects of genistein in the experimental system used in this work has been demonstrated in MTT and Annexin V assays (Fig. 3).

One should also note that it was demonstrated recently that in 3-nitropropionic acid (3-NPA)-treated rats, genistein improves memory impairment (Menze et al. 2015) and sensorimotor gating (Menze et al. 2016). 3-NPA is a mitochondrial toxin that causes lesions in the brain which are similar to that found in HD. Therefore, the 3-NPA-induced animal model reflects only secondary effects of HD (increased level of the brain damage, increased level of reactive oxygen species, energetic disturbances); however, it does not induce the primary cause of this disease, i.e., appearance of mutated huntingtin aggregates. Due to these restrictions, studies on genistein action in this model might only indicate its effects on HD symptoms, with no information about the primary cause. On the contrary, we have demonstrated that genistein corrects the HD phenotype thorough stimulation of the autophagy process and reduction of the primary cause of this disease.

In conclusion, results presented in this report, obtained in studies on the cellular model of HD (transfected HEK-293 cells), indicated that treatment with genistein can correct the storage of mutated huntingtin aggregates (Figs. 1, 2) and improve cell viability (Fig. 3). We recognize these results encouraging, as they provide the basis for further experiments on both molecular mechanisms of genistein-mediated correction of HD and development of an efficient therapy for this inherited neurodegenerative disease. Obtaining such indicative results on cellular models of other genetic diseases has been exemplified in research leading to development of promising therapies, including that based on the use of genistein for treatment of another genetic neurodegenerative disorder, Sanfilippo disease, which after original demonstration of the effects in cell cultures (Piotrowska et al. 2006), reached clinical trials (de Ruijter et al. 2012; Kim et al. 2013) that are now at the level of phase III (EudraCT number: 2013-001479-18).
